# Decreased expression of Glucagon-like peptide-1 receptor and Sodium-glucose co-transporter 2 in patients with proliferative diabetic retinopathy

**DOI:** 10.3389/fendo.2022.1020252

**Published:** 2022-11-17

**Authors:** Hui Chen, Xiongze Zhang, Nanying Liao, Yuying Ji, Lan Mi, Yuhong Gan, Yongyue Su, Feng Wen

**Affiliations:** State Key Laboratory of Ophthalmology, Zhongshan Ophthalmic Center, Sun Yat-sen University, Guangdong Provincial Key Laboratory of Ophthalmology and Visual Science, Guangzhou, China

**Keywords:** diabetic retinopathy, glucagon-like peptide-1 receptor, sodium-glucose co-transporter, glucose transporter, cytokines

## Abstract

**Purpose:**

To investigate the expression of Glucagon-like peptide-1 receptor (GLP-1R), sodium-glucose co-transporter (SGLT) 1, SGLT2, Glucose transporter type 1 (GLUT1) and GLUT2 in patients with diabetic retinopathy (DR).

**Methods:**

We obtained peripheral blood mononuclear cells (PBMCs) and vitreous samples from 26 proliferative DR (PDR) patients, 25 non-proliferative DR (NPDR) patients, 25 non-DR (NDR) patients, and 26 nondiabetic patients with idiopathic epiretinal membranes (ERMs, control). The protein level and mRNA expression level of GLP-1R were quantified by immunoblot and qRT-PCR and the levels of SGLT1, SGLT2, GLUT1, and GLUT2 expression were determined by PCR. Their association with clinical parameters and PBMCs/vitreous cytokine was analyzed. Furthermore, immunofluorescence staining of GLP-1R and SGLT2 was carried out on samples of fibrovascular membranes (FVMs) retrieved from 26 patients with PDR and 26 patients with ERMs.

**Results:**

The transcriptional levels of GLP-1R and SGLT2 in PBMCs were significantly more decreased in PDR patients than in patients without DR and controls, which was simultaneously associated with an increased level of expression of tumor necrosis factor (TNF)-α and interferon (IFN)-γ. The expression levels of GLUT1 and GLUT2 were tightly correlated with their SGLT partners, respectively. Further, Immunofluorescence staining showed no positive staining of GLP-1R and SGLT2 was detected in the FVMs from PDR.

**Conclusions:**

GLP-1R and SGLT2 were significantly decreased in PDR patients which was associated with an increased level of expression of TNF-α and IFN-γ. These findings implicate that defective GLP-1R and SGLT2 signaling may potentially correlate with immune response cytokines in patients with PDR.

## Introduction

Diabetic retinopathy (DR) is a diabetic microangiopathies commonly occurred as a complication of type 2 diabetes mellitus (T2DM), and the most common cause of sight-threatening blindness worldwide (1). The molecular mechanisms underlying this disease are therefore highly demanded for the development of novel treatment strategies. Intensive studies has been focused on the use of non-insulin anti-hyperglycaemic agents, including agonists of glucagon-like peptide-1 receptor (GLP-1R) and inhibitors of sodium-glucose co-transporter-2 (SGLT-2), in the treatments of T2DM.

Glucagon-like peptide-1 (GLP-1) has becoming a special interest as a treatment target due to its broad regulatory roles in maintaining glucose homeostasis. GLP-1 is postprandially secreted by intestinal enteroendocrine L-cells, and enhances the glucose-induced insulin release from pancreatic beta-cells (2). The G-protein-coupled membrane receptor, GLP-1R, has also been discovered in the pancreatic islets’ cells and in various other kinds of tissues as well, including the kidney, heart, blood vessels, central nervous system, and retina (Lin et al., 2018; Shi et al., 2015). On the other hand, several glucose sensors, such as electrogenic glucose transport SGLT 1/SGLT 2 and facilitative glucose transporter (GLUT)1/GLUT 2, have been suggested to associate with the glucose-exposure-induced GLP-1 secretions. It has been proposed that glucose induces GLP-1 release through SGLT1/SGLT 2, and to a lesser extent, GLUT 1/GLUT 2.

At the same time, anti-hyperglycaemic agents are demonstrated to produce protective or neutral influences on eye complications of diabetes. Previous studies have reported the potential beneficial influences of GLP-1 agonists in the treatments of diabetic retina through the functional improvements of blood retina barrier and the inhibition of neuronal apoptosis (3). Study on spontaneously diabetic fatty rats reveals that alleviation of hyperglycaemia by the treatment of SGLT-2 inhibitor is able to limit the development of microvascular complications of diabetes such as diabetic retinopathy (4).

However, underlying mechanisms of the protective effects of anti-hyperglycaemic agents on DR patients remains unclear. Currently, immunity dysregulation is considered as a significant pathogenic mechanism in DR, both locally as well as systematically (5). Metabolic imbalance concerning glucose metabolism potentially results in a dysregulation in the function and dissemination of T-lymphocytes, leading to dysfunctional cell-mediated immune responses, which is considered as a contributor to the pathogenesis of DR (6). In addition, GLP-1Rs have been detected on immune cells, and its anti-inflammatory effects include the inhibition of TNF-α (7). Therefore, it appears feasible to speculate that GLP-1/GLP-1R signaling is related to the functions of T-lymphocytes in DR patients.

In the present study, we quantified the expression levels of GLP-1R, SGLT1, SGLT2, and the respective cognate basolateral transporters (GLUT1 and GLUT2) in peripheral blood mononuclear cells (PBMCs). The pro-inflammatory cytokines associated with T helper cells including TNF-α, and IFN-γ in PBMCs and vitreous fluid were also measured. Subsequently, these results were further confirmed in tissue samples obtained from patients with proliferative DR (PDR).

## Materials and methods

### Patients

Twenty-six PDR patients, 25 non-proliferative diabetic retinopathy (NPDR) patients, 25 non-DR (NDR) patients, and 26 nondiabetic patients with idiopathic epiretinal membranes (ERMs) who received vitrectomy were recruited from the Zhongshan Ophthalmic Center between Jan and July 2021 (**Table 1**). Patients with a medical history of intraocular surgery, ocular trauma, ocular inflammatory diseases, trauma, vitreous hemorrhage, uveitis, retinal detachment, systemic or topical steroid treatment, and immunosuppressive drug administration were excluded. All diagnoses were carried out according to the 2002 standards of the American Diabetes Association (8). Exclusion criteria included infectious disease, diabetes-associated nephropathy (including patients with chronic kidney disease in stage 3, proteinuria, and macroalbuminuria, and patients receiving hemodialysis) and patients who received intraocular or intravitreal treatments and photocoagulation within 3 months upon recruitment. Chronic kidney diseases were categorized according to the clinical guidelines of the National Kidney Foundation Disease Outcomes Quality Initiative. DR was diagnosed according to the results of fluorescein fundus angiography (FF450 fundus camera; Carl Zeiss, Germany). Furthermore, the Body mass index (BMI) of patients was also collected. All recruited patients were subcategorized into three groups based on the Diabetic Retinopathy Disease Severity Scale: NDR, NPDR, and PDR (9).

**Table 1:** Clinical and biochemical characteristics of type 2 diabetic patients and healthy control subjects.

	Control(N = 26)	NDR(N =25)	NPDR(N = 25)	PDR(N = 26)	p
Sex(m/f)	13/13	12/13	11/14	14/12	0.916
Age(years)	62.8 ± 6.9	64.3 ± 8.7	61.6 ± 8.1	63.7 ± 6.0	0. 623
BMI(kg/m2)	22.5 ± 2.2	23.0 ± 2.5	23.4 ± 2.2	25.1 ± 4.4	0.011
Diabetes Duration(years)	-	8.2 ± 3.4	9.7 ± 3.0	14.0 ± 2.0	<0.001*
FPG(mmol/l)	5.3 ± 0.7	7.8 ± 1.6	9.6 ± 2.1	12.4 ± 1.8	<0.001*
HbAlc(%)	5.1 ± 0.7	7.3 ± 1.4	8.8 ± 1.9	11.4 ± 1.8	<0.001*

DR, diabetic retinopathy; NDR, no apparent retinopathy; NPDR, non-proliferative retinopathy; PDR, proliferative diabetic retinopathy; BMI, Body mass index; FPG, fasting plasma glucose; HbA1c, glycated hemoglobin.

Data are expressed as mean ± SD.

^*^ P ≤ 0.05.

All experimental procedures were carried out following the principles of the Declaration of Helsinki, and authorized by the Human Ethics Committee of Zhongshan Ophthalmic Center of Sun Yat-sen University. Each included patient was fully informed and written informed consents were obtained.

### Sample preparation

Whole blood specimens (12 mL, with anticoagulant lithium heparin) were collected from all recruited patients and healthy controls for isolation of PBMCs, protein and mRNA expression tests. Blood sample aliquots were also obtained to conduct fasting plasma glucose (FPG) and glycated hemoglobin (HbA1c) tests.

### PBMCs isolation

PBMCs were extracted from heparinized blood samples through Ficoll-Hypaque density gradient centrifugations (Lymphoprep; Nycomed Pharma, Norway). PBMCs (2 × 106 cells/ml) were stimulated with phytohaemagglutinin (PHA) to assess the production of TNF-α and IFN-γ. Isolated PBMCs were stimulated for 48h, and subsequently used for TNF-α and IFN-γ analysis by ELISA.

### Vitreous fluid

During pars plana vitrectomy, samples containing undiluted vitreous fluid (0.5 ml) were obtained from 26 PDR patients, 25 NPDR patients, 25 NDR patients, and 26 nondiabetic patients with ERMs. All the samples were preserved at −80°C until they were needed for further analyses.

### RNA extraction and quantitative real-time PCR

TRIzol reagent (Carlsbad, USA) was utilized to extract the total RNA of PBMCs and a reverse transcription kit (Toyobo, Japan) was used for reverse transcription to cDNA. qRT-PCR was conducted on a LightCycler CFX96 (BioRad, USA) using QuantiFast SYBR Green PCR Kit (Qiagen, Germany). The primers that were used in this study are described as follows: GLP-1R forward: 5’-GTT TCA TGA TGG CCT GAG GT-3’, reverse: 5’-CTG ACT ACT GAA TTG GAA GGG G-3’; SGLT1 forward: 5’-CTC CCT TTC TTA TTC TCC CAG GAT-3’, reverse: 5’-GCC CAG GAG ATC AAG GCT ATA GTA-3’; SGLT2 forward: 5’-ATA AAC AGC TGG GCT GTC CC-3’, reverse: 5’-CGT AAC CCA TGA GGA TGC AG-3’; GLUT1 forward: 5’-AGG GCT GGA GTG AGG GTA GT-3’, reverse: 5’-CAT ACA TCT GTG GGG CAG C-3’; GLUT2 forward: 5’-AAA CAA AGC AAA TGT TCA GTG G-3’, reverse: 5’-TGG GTC CCC AAA AGC TTA G-3’; TNF-α forward5′-CCCAGGCAGTCAGATCATCTTC-3′.Reverse:5′-AGCTGCCCCTCAGCTTGA-3′.; IFN-γ forward: 5’-TCAACTTCTTTGGCTTAATTCTCTC-3’, reverse: 5’-ATATGGGTCCTGGCAGTAACA-3’ and β-actin forward: 5’-GGA CTT CGA GCA AGA GAT GG-3’, reverse: 5’-AGC ACT GTG TTG GCG TAC AG-3’. β-actin acted as an internal control. All samples were tested in triplicates. The single peak in the melting curve was used for primer specificity confirmations. The relative mRNA expression levels were estimated according to the ΔΔCt method.

### Immunoblotting

Protein samples of PBMCs isolated from T2DM patients as well as healthy controls were prepared in RIPA buffer. Aliquots of 60 μg protein were divided by 12% sodium dodecyl sulfate-polyacrylamide gel electrophoresis (SDS-PAGE) and placed on polyvinylidene fluoride (PVDF) membranes *via* semidry electroblotting. The primary antibodies GLP-1R (Abcam, UK) and β-actin (Abcam, UK) were used for immunoblotting: Immunoblot was visualized on radiographic films using the SuperSignal West Pico Substrate Kit (Pierce, USA), and the software Image J (National Institutes of Health, USA) was applied for analysis. β-actin served as internal control.

### Cytokine ELISA

The concentration of TNF-α and IFN-γ in the supernatants of collected PBMCs and vitreous fluid were determined by DuoSet ELISA kits (R&D Systems) as instructed by the manufacturer. The lowest detectable concentration of TNF-α was 15.6 pg/ml and 9.4 pg/ml for IFN-γ. These measurements were performed in duplicate.

### Immunofluorescence staining of FVMs

The fibrovascular membranes (FVMs) of T2DM patients with PDR (26 cases) were surgically detached through membrane peeling during pars plana vitrectomy. ERM resections were carried out on 26 idiopathic ERM patients as control. As **Table 1** shows, significant differences in age and gender were not detected between the groups.

Samples of ERMs were embedded in an ideal cutting compound, fast frozen, and preserved at -80°C within 1h following collection of the fresh samples. The following primary antibodies were used for immunofluorescence staining: anti-GLP-1R polyclonal IgG (Abcam, ab214185, 1:300) and anti-SGLT2 polyclonal IgG (Abcam, ab180799, 1:200). DAPI (Sigma-Aldrich, D9542, 1:1000) was used for visualization of the nuclear morphology. Immunofluorescence staining was examined and images were captured under a fluorescence microscope (DS-Ril-U2; Nikon, Japan)

### Statistical analysis

SPSS software (version 22.0, SPSS Inc., USA) was used to carry out statistical analysis. Nonparametric Kruskal-Wallis tests or One-Way Analysis of Variance (ANOVA) was conducted for the group variation analysis between T2DM patients and healthy controls. Mann-Whitney U tests or t-tests were used for the analysis between each group. Spearman’s correlation tests were used to establish potential correlations between parameters. The multivariable models of GLP-1R and SGLT2 were utilized to better understand their clinical implications in relation to DR, BMI, diabetes duration, FPG, HbAlc, age and sex. All graphs were generated by GraphPad Prism version 5 and the data were expressed as mean ± SD. P < 0.05 was set as the cut-off for statistical significance.

## Results

### Clinical features

Statistically significant differences (P =0.623) were not found in the average age of T2DM patients (76 patients, of which 37 male and 39 female, average age 63.2 ± 7.6 years old) and normal controls (26 patients, of which 13 male and 13 female, average age 62.8 ± 6.9 years-old), as shown in **Table 1**. All T2DM patients were divided in either one of the following three groups: NDR (n=25), NPDR (n=25), and PDR (n=26). As a result, the male/female ratios and average ages of each group were as follows: NDR: 12/13, 64.3 ± 8.7 years old; NPDR: 11/14, 61.6 ± 8.1 years old; and PDR: 14/12, 63.7 ± 6.0 years old.In addition, statistically significant differences in gender were also not detected among the groups (P = 0.916). However, a significantly higher BMI was found in T2DM patients than healthy controls (P = 0.011). A significantly longer course of disease was identified in PDR patients in comparison to NPDR and NDR patients (P < 0.001). Statistically significant higher HbA1c and FPG levels were found in PDR patients in comparison to NPDR (P < 0.001) and NDR (P < 0.001) patients.

### mRNA expression levels of GLP-1R, SGLT1, SGLT2, GLUT1, GLUT2, TNF-α and IFN-γ

**Figure 1:**
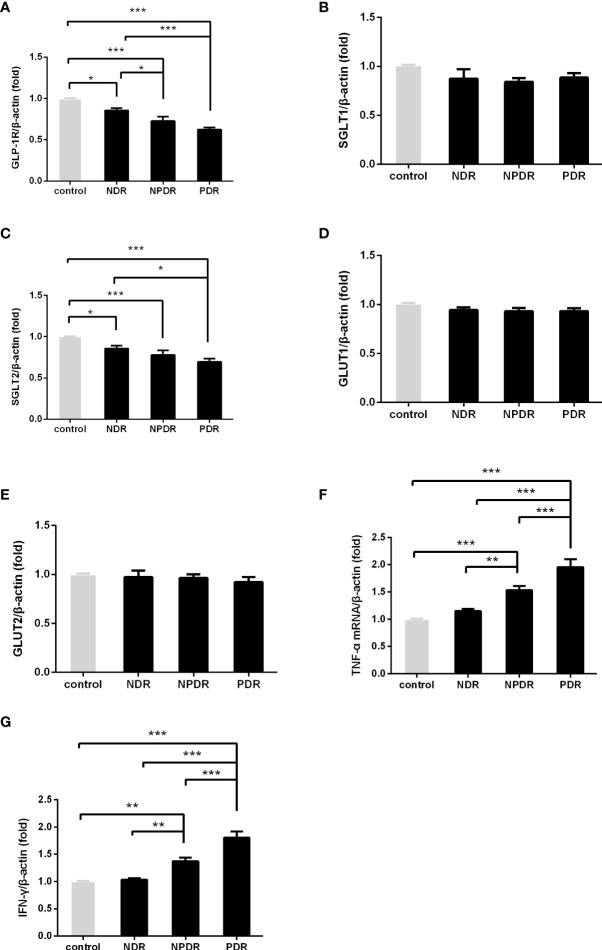
The mRNA expression of GLP-1R and SGLT2 was decreased and that of TNF-α and IFN-γ was elevated in DR patients. The mRNA expression of GLP-1R, SGLT1, SGLT2, GULT1, GULT2, TNF-α and IFN-γ in freshly obtained PBMCs was quantified by real-time PCR and normalized to the expression levels of β-actin. (PDR, n=26; NPDR, n=25; NDR, n=25; control, n=26) **(A–G)**. The values represent the fold-change in comparison to the controls. *P < 0.05, **P < 0.01, ***P < 0.001.

**Figure 2:**
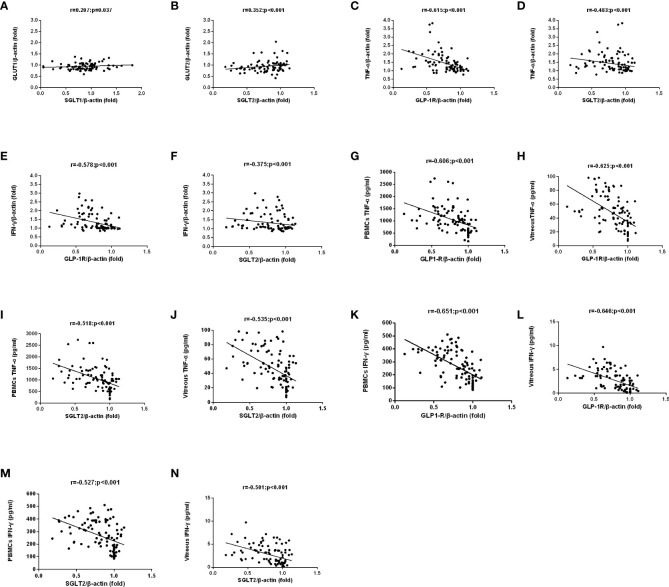
The correlation between SGLTs and GLUTs was analyzed in matched samples with the Spearman correlation. GLUT2 and GLUT1 expression were closely correlated to those of the corresponding SGLT partners. **(A, B)**. A negative correlation among TNF-α/IFN-γ mRNA and protein expression and GLP-1R/SGLT2 mRNA expression in PBMCs of all patients were found **(C–N)**.

We investigated the mRNA levels of GLP-1R, SGLT1, SGLT2, GLUT1, GLUT2, TNF-α, and IFN-γ in PBMCs isolated from T2DM patients and healthy controls using qRT-PCR (**Figure 1**). The findings indicated significantly lower mRNA expression levels of GLP-1R (both P < 0.001) and SGLT2 (P = 0.021 and P < 0.001, respectively) in PBMCs isolated from PDR patients than that of NDR patients and healthy controls. On the contrary, the mRNA expression levels of TNF-α (both P < 0.001) and IFN-γ (both P < 0.001) were significantly higher in PDR patients in comparison to NDR patients and controls. Meanwhile, we also found a significant intercorrelation between the expression of SGLT1 and GLUT1, and also between SGLT2 and GLUT2 (**Figures 2A, B**). However, the differences in the expression ratios of SGLT1/GLUT1 (P = 0.622) and SGLT2/GLUT2 (P = 0.087) were not significant among different groups of patients.

**mRNA Levels of GLP-1R and SGLT2 and demographic factors**

**Figure 3:**
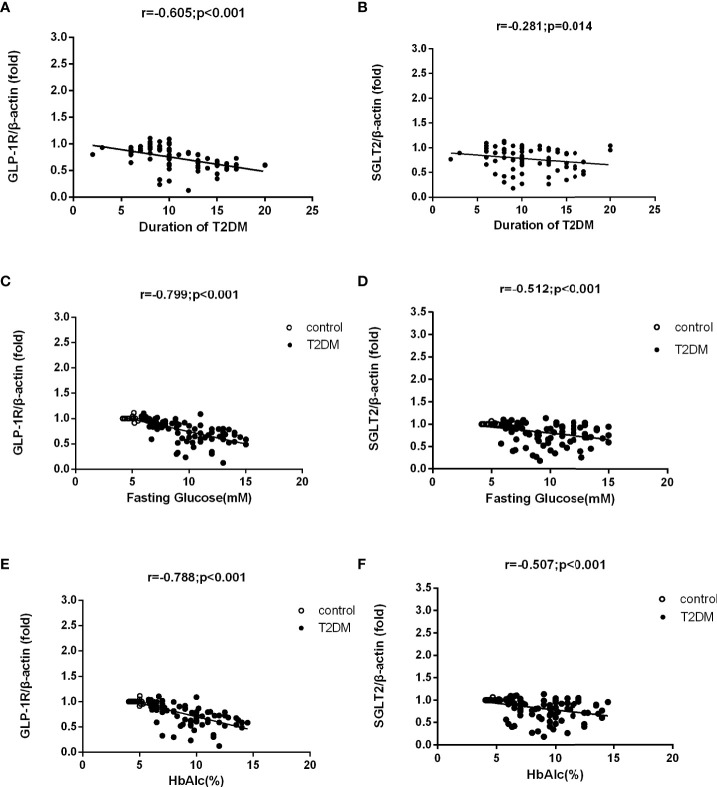
Correlation analysis of mRNA levels of GLP-1R and SGLT2 and demographic factors in T2DM patients and control group. mRNA levels of GLP-1R and SGLT2 were both negatively correlated to the course of disease in T2DM patients **(A, B)**. The mRNA levels of GLP-1R and SGLT2 were also negatively correlated to the levels of FPG and HbA1c in T2DM patients and the control group **(C–F)**.

**Figure 3** shows that the detected levels of GLP-1R (r = -0.605, P < 0.001) and SGLT2 mRNA (r = -0.281, P =0.014) both had a negative correlation with the course of disease in T2DM patients. Meanwhile, we also discovered that the mRNA levels of GLP-1R (r = -0.799, P < 0.001 and r = -0.788, P < 0.001, respectively) and SGLT2 (r = -0.512, P < 0.001 and r = -0.507, P < 0.001, respectively) indicated a negative correlation with the levels of FPG and HbA1c as well.

### GLP-1R protein levels in PBMCs

**Figure 4:**
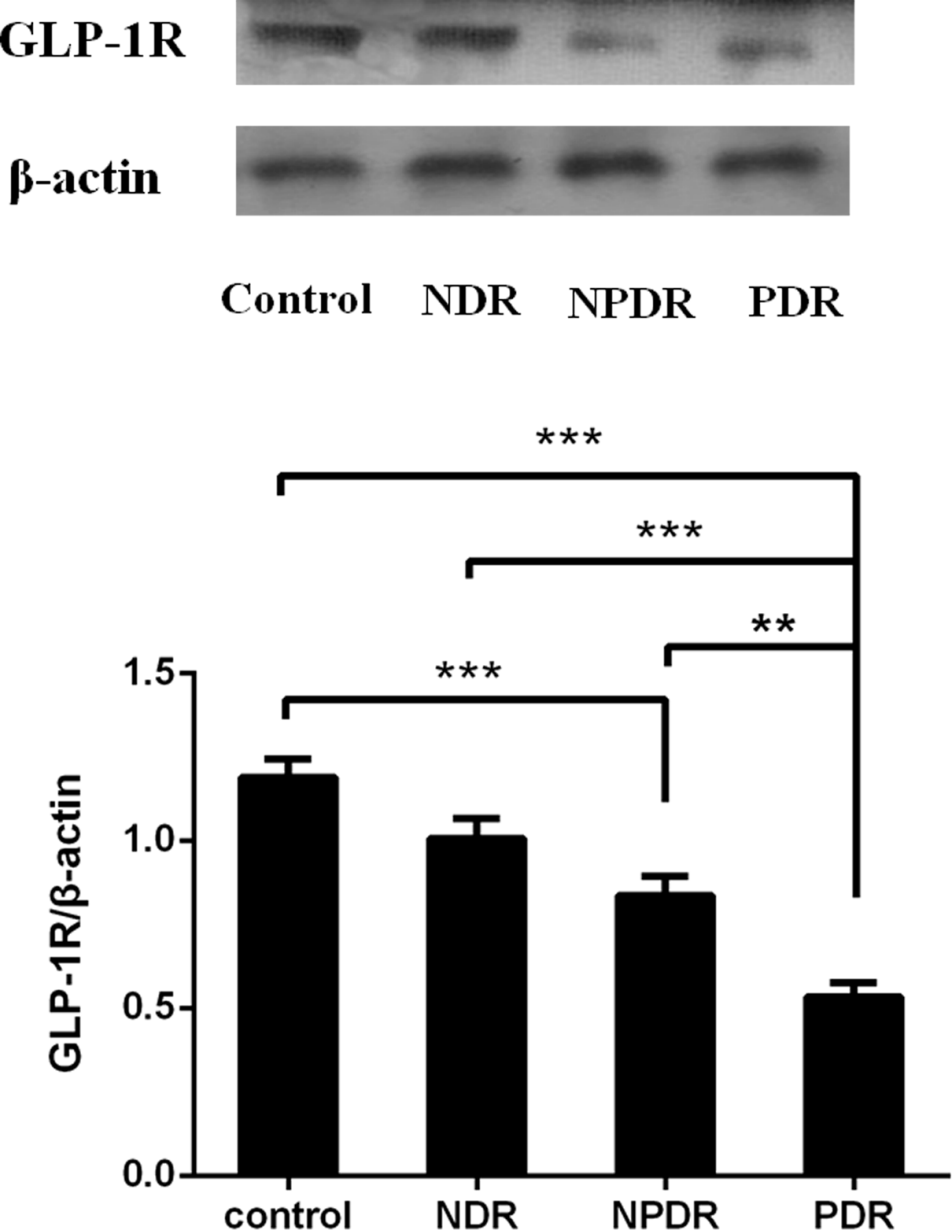
The protein expression of GLP-1R was decreased in DR patients (PDR: n=26; NPDR: n=25; NDR: n=25; control: n=26). Western blot analysis (lane 1, healthy control; lane 2, NDR; lane 3, NPDR; and lane 4, PDR) and quantitation of GLP-1R from PBMCs. β-actin was applied as the internal control. **P < 0.01, ***P < 0.001.

For further verification of the downregulation trend of GLP-1R in DR patients, we tested the protein levels of GLP-1R in the PBMCs isolated from DR patients before receiving any clinical treatments and healthy controls. As shown in **Figure 4**, we found significantly decreased protein levels of GLP-1R in PDR patients in comparison to both NDR patients (P < 0.001) as well as normal controls (P < 0.001). Moreover, the protein levels and mRNA expression levels of GLP-1R in every group were significantly positively correlated (r = 0.604; P < 0.001).

**Multivariate regression analysis of GLP-1R and SGLT2 as dependent variable in the T2D samples**

Multiple linear regression analysis revealed that PDR remained independently and negatively associated with GLP-1R protein level and SGLT2 mRNA level after adjustment for age, gender, BMI, diabetes duration, FPG and HbAlc (P=0.003 and P=0.028, respectively). Furthermore, in this model, diabetes duration was found independently and negatively associated with GLP-1R mRNA expression (P=0.028) (**Table 2**).

**Table 2:** Multivariate Regression Analysis With GLP-1R and SGLT2 as Dependent Variable in T2D samples.

Independent Variables	GLP-1R mRNA		GLP-1R protein		SGLT2 mRNA	
	β (95 %CI)	*P*	β (95 %CI)	*P*	β (95 %CI)	*P*
Groups						
NPDR PDR	-0.046 (-0.147,0.055)-0.011 (-0.138,0.160)	0.3690.881	-0.128 (-0.2821,0.027) -0.341 (-0.569,-0.113)	0.1060.003*	-0.104 (-0.236,0.027) -0.217 (-0.411,-0.023)	0.1190.028*
Age	0.002 (-0.004,0.007)	0.553	0.000 (-0.008,0.008)	0.978	0.001 (-0.006,0.007)	0.876
Sex	0.011 (-0.067,0.089)	0.774	-0.040 (-0.160,0.079)	0.510	0.008 (-0.093,0.109)	0.877
BMI	0.001 (-0.012,0.013)	0.908	-0.010 (-0.029,0.010)	0.3175	0.003 (-0.014,0.019)	0.727
Diabetes duration	-0.016 (-0.030,0.002)	0.028*	-0.011 (-0.033,0.011)	0.339	-0.007 (-0.025,0.012)	0.476
FPG	-0.017 (-0.074,0.039)	0.551	-0.018 (-0.104,0.069)	0.686	0.026 (-0.048,0.099)	0.493
HbAlc	-0.017 (-0.078,0.044)	0.589	0.008 (-0.086,0.102)	0.868	-0.007 (-0.087,0.073)	0.867

T2D, Type 2 diabetes; NPDR, non-proliferative retinopathy; PDR, proliferative diabetic retinopathy; BMI, Body mass index; FPG, fasting plasma glucose; HbA1c, glycated hemoglobin.

^*^P ≤ 0.05

### Vitreous cytokines

**Figure 5:**
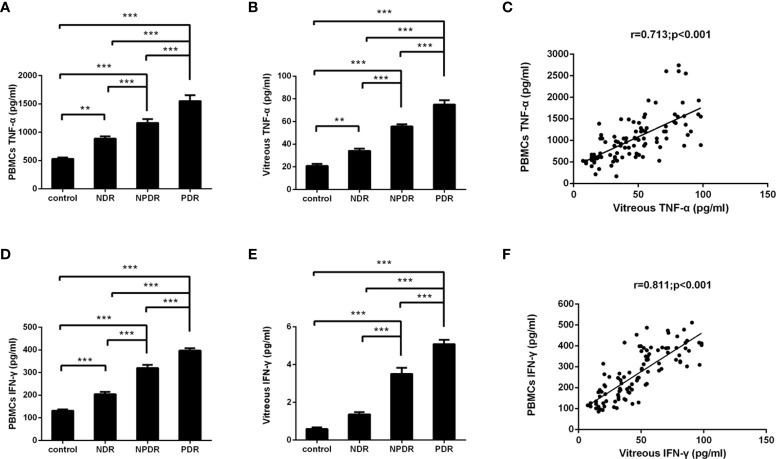
Concentrations of TNF-α/IFN-γ in PBMCs supernatants and vitreous fluid of T2DM patients and non-diabetic controls. TNF-α and IFN-γ were measured with ELISA in PBMCs supernatants **(A, D)** and vitreous fluid **(B, E)** of controls (n=26), non-diabetic retinopathy (NDR, n=25), nonproliferative diabetic retinopathy (NPDR, n=25), and proliferative diabetic retinopathy (PDR, n=26). Between group comparisons were conducted with the Kruskal–Wallis test and then the Dunn multiple comparison test. The correlation between concentrations of TNF-α/IFN-γ in the PBMCs supernatants as well as vitreous fluid was analyzed in matched samples with the Spearman correlation **(C, F)**. **P < 0.01, ***P < 0.001.

The PBMCs and vitreous concentrations of TNF-α and IFN-γ according to DR status are shown in **Figure 5**. The detected levels of TNF-α and IFN-γ in the PBMCs and vitreous fluid of PDR patients (all P < 0.001) were statistically significantly enhanced in comparison to those of NDR patients and healthy controls. In addition, statistically significant correlations were found among the concentrations of TNF-α (r = 0.713, P < 0.001) and IFN-γ (r = 0.811, P < 0.001) in PBMCs and vitreous fluid (**Figure 5C, F**).

### Correlation between GLP-1R/SGLT2 expression and TNF-α/IFN-γ expression

A negative correlation was found between the mRNA levels of GLP-1R and SGLT2 in PBMCs on the one hand and mRNA levels of TNF-α and IFN-γ on the other (**Figure 2C–F**). Meanwhile, negative correlations were also discovered between the mRNA levels of GLP-1R and SGLT2 on one hand and TNF-α and IFN-γ on the other in PBMCs and vitreous fluid (**Figure 2G–N**). These findings show that the expression of GLP-1R and SGLT2 is correlated to TNF-α/IFN-γ Expression.

### GLP-1R and SGLT2 Expression in FVMs of PDR patients

**Figure 6:**
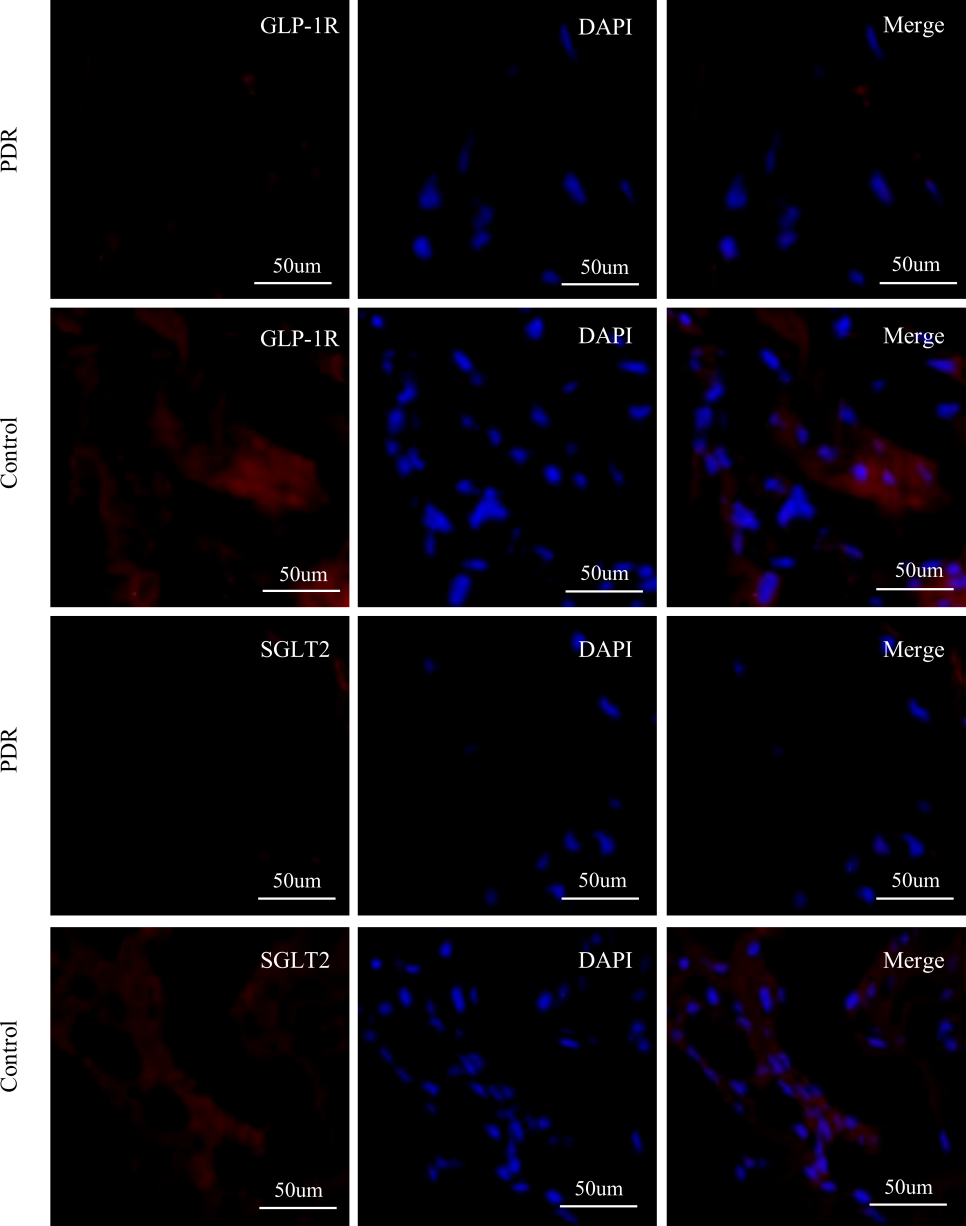
Immunofluorescence staining of GLP-1R, SGLT2, and DAPI in fibrovascular membranes of PDR patients. The staining reaction of GLP-1R and SGLT2 (red) was clearly positive on the ERM of a patient of the control group. The DAPI stain (blue) showed numerous nuclei. No GLP-1R-positive reactions and SGLT2-positive are detected on the FVM from PDR patient. Scale bar: 50 μm.

In our experiments, positive staining of GLP-1R in the FVMs collected from PDR patients was not identified. Furthermore, positive staining of SGLT2 was also not detected in the membranes collected from PDR patients (**Figure 6**).

## Discussion

In this study, we provided evidence for the changes in GLP-1R expression levels in DR patients. Moreover, we obtained the quantitative expressions of SGLT1/SGLT2 and GLUT1/GLUT2 in DR patients. Our results have indicated that PDR patients were characterized by decreased GLP-1R and SGLT2 expression, which was related to a higher expression of TNF-α and IFN-γ. The expressions of GLP-1R and SGLT2 were subsequently confirmed in the FVM collected from the PDR patients by immunofluorescence. Additionally, we found that the expression ratios of SGLT1/GLUT1 and SGLT2/GLUT2 were similar; meanwhile, both the expression levels of SGLTs showed significant correlations with the expression of respective GLUTs genes. These results are in agreement with the finding that SGLTs interconnect to specific isoforms of GLUTs (10).

In this study, we found that the level of GLP-1R was significantly reduced in PBMCs isolated from PDR patients. We also found that the levels of GLP-1R in eye samples collected from PDR patients in advanced stages was not detectable, which was consisted with previous study (11). As a gut incretin hormone, GLP-1 is produced in intestine by L cells. GLP-1 participates in the regulation of glucose homeostasis through stimulating insulin secretions and suppressing glucagon releasing in response to glucose intakes (12). Meanwhile, GLP-1 also produces functions glycemic independently in different organs (13). The effects produced by GLP-1 in is reflected by the local activations of GLP-1R (14). The expression of GLP-1R in retinal pericytes and ganglion cells was previously demonstrated (15), while GLP-1/GLP-1R was also reported to produce beneficial effects under the condition of hyperglycemia (16), suggesting that GLP-1/GLP-1R has protective effects on the integrity of retina in the first phases of DR caused by diabetes (3). Consistently, one previous study demonstrated the neuroprotective abilities of GLP-1R agonists in DR of db/db mice (17). Nonetheless, the possible mechanisms responsible for its protective effects are still unknown.

We found a positive correlation between TNF-α and IFN-γ production and expression levels of GLP-1R, which were consistent with the observation that modulation in GLP-1R signaling control host microbial responses and innate immune responses in a mouse model (18).

An accumulating amount of studies have been reporting about the immune dysfunction of T cells in DR (6, 19). A continuous decline in T cell function can be caused by sustained signaling in DR, and it has been well established that TNF-α, IFN-γ, and their distinct receptors are vital components of the innate immune system. Consistent with these outcomes, we also discovered higher expressions of TNF-α and IFN-γ mRNA in PBMCs of DR patients compared to that of non-DR subjects, which simultaneously showed a higher incidence of increased TNF-α and IFN-γ production in correlation to the progression of DR severity.

In our study, we further assessed the role of SGLT1, SGLT2, GLUT1, and GLUT2 in GLP-1 release. GLUTs mainly consist of GLUTs, such as GLUT5, GLUT7, GLUT9, and GLUT11, and SGLTs (10, 20, 21). Of the SGLTs family, subtypes SGLT1 and SGLT2 have been intensively studied. Inhibitors of SGLT2 have already been applied in the clinical treatments of patients with diabetes (22). It is well known that SGLTs and GLUTs produce active and facilitative effects, respectively. The relative roles of SGLTs and GLUTs in GLP-1 secretion induced by glucose have been investigated in seminal studies using pharmacological and genetic interference with SGLTs and GLUTs (23–25), suggesting that they were essential for GLP-1 secretion induced by glucose associated with the cAMP and Ca2+ signaling system (26, 27). Consistent with studies linking decreased SGLT activity with reduced GLP-1, our study found that GLP-1R and SGLT2 were simultaneously decreased in PDR patients (28), these data indicated that release of GLP-1in DR might be a process that requires SGLT2-mediated glucose transport in the signal transduction pathway.

Despite the lack of understanding of the regulation in this process, evidence derived from mouse models and preclinical and clinical research suggested that SGLT2 inhibitors produced effects that reduced tissue inflammation (29–32). A recent study also reported that SGLTs are absent in retinal endothelial cells (33). Meanwhile, no clear evidence has been reported demonstrating SGLT2 expression human retina cells. In the present study, we provided evidence showing the potential function and expression of SGLT2 in DR patients for the first time. Recent studies also demonstrated the downregulation of both SGLT2 and GLUT2 in T2DM patients (34). However, conflicting results also showed upregulated SGLT2 expression in patients with kidney diseases related to diabetes (35). The contrasting results in those clinical studies were potentially caused by multiple reasons, such as different techniques in collecting human tissue and inclusion of different human races, populations, and T2DM patient population. For this reason, further studies are necessary to explain the regulations of SGLTs in DR.

We also found that the expression of GLUTs was reduced in PBMCs isolated from PDR patients compared to healthy controls. However, this change was not statistically significant and entirely proportionate to the changes in the expressions of SGLTs. As these two types of transporters were anatomically linked, the covariance in the changes of expressions was further proved in our study. In recent studies of the oxidative stress-related inflammation responses caused by hyperglycemia, the downregulated expression of GLUT1 in the retina was found to be correlated to the reduced GLUT1 level on cell membranes due to subcellular redistribution (36, 37). Previous research has also identified the reduction of GLUT1 in retina cells in streptozotocin-induced diabetes in rats (38), and the inhibition of GLUT1 protein translations on the blood-brain barrier (BBB) in diabetes (39).

Finally, we provided evidence indicating negative correlations among disease duration of T2DM, FPG, HbA1c and mRNA levels of GLP-1R and SGLT2 in DR patients, which were consistent with previous studies of animal models in addition to T2DM patients (34, 40). The results above imply that the duration of disease in diabetes and the degree of glycemic maintenance are of critical importance in diabetes treatment and the prevention of related complications.

Our study has some limitations, including its observational design and the proportionately small sample size. As retinal vascular abnormalities are prevalent comorbidities of DR, and GLP-1R analogs have already been used clinically in diabetes and obesity, further pre- and clinical research is necessary to elucidate the regulative mechanism underlying GLP-1R/SGLT2 signaling in DR.

In conclusion, we investigated the role of glucose sensors, including SGLT1, SGLT2, GLUT1, GLUT2, and GLP-1R in patients with DR in the current study. Our results from PBMCs and FVM demonstrated that GLP-1R and SGLT2 were less expressed in PDR patients than in healthy controls, which was associated with increased TNF-α and IFN-γ production. These outcomes suggest that the restoration of GLP-1R/SGLT2 signaling is potentially involved in regulation of immune checkpoint molecules in DR patients. However, it is still unknown if GLP-1R/SGLT2 signaling and GLP-1R analogs could be used as potential immunomodulatory targets in DR therapy. Further prospective studies are imperative to elucidate the influence of GLP-1R/SGLT2 signaling on the progression of DR.

## Data availability statement

The original contributions presented in the study are included in the article/supplementary material. Further inquiries can be directed to the corresponding author.

## Ethics statement

The studies involving human participants were reviewed and approved by Human Ethics Committee of Zhongshan Ophthalmic Center of Sun Yat-sen University. The patients/participants provided their written informed consent to participate in this study.

## Author contributions

HC and FW contributed to the conception of the study; XZ, NL,YG and YS performed the experiment; NL, LM contributed significantly to analysis and manuscript preparation; HC performed the data analyses and wrote the manuscript; XZ helped perform the analysis with constructive discussions. All authors contributed to the article and approved the submitted version.

## Funding

This study was supported by the National Natural Science Foundation of China (81970813), the Natural Science Foundation of Guangdong Province (2018A030313635) and Guangzhou Municipal Science and Technology Project (201904010062)

## Conflict of interest

The authors declare that the research was conducted in the absence of any commercial or financial relationships that could be construed as a potential conflict of interest.

## Publisher’s note

All claims expressed in this article are solely those of the authors and do not necessarily represent those of their affiliated organizations, or those of the publisher, the editors and the reviewers. Any product that may be evaluated in this article, or claim that may be made by its manufacturer, is not guaranteed or endorsed by the publisher.

## References

[1] Liew G Wong VW Ho IV. Mini review: Changes in the incidence of and progression to proliferative and sight-threatening diabetic retinopathy over the last 30 years. Ophthalmic Epidemiol (2017) 24:73–80. doi: 10.1080/09286586.2016.1259638 28102748

[2] Doyle ME Egan JM. Mechanisms of action of glucagon-like peptide 1 in the pancreas. Pharmacol Ther (2007) 113:546–93. doi: 10.1016/j.pharmthera.2006.11.007 PMC1934514 17306374

[3] Pang B Zhou H Kuang H. The potential benefits of glucagon-like peptide-1 receptor agonists for diabetic retinopathy. Peptides (2018) 100:123–6. doi: 10.1016/j.peptides.2017.08.003 28807775

[4] Takakura S Toyoshi T Hayashizaki Y Takasu T. Effect of ipragliflozin, an SGLT2 inhibitor, on progression of diabetic microvascular complications in spontaneously diabetic torii fatty rats. Life Sci (2016) 147:125–31. doi: 10.1016/j.lfs.2016.01.042 26829386

[5] Rubsam A Parikh S Fort PE. Role of inflammation in diabetic retinopathy. Int J Mol Sci 19 (2018) 19:942. doi: 10.3390/ijms19040942 PMC5979417 29565290

[6] Chen H Wen F Zhang X Su SB. Expression of T-helper-associated cytokines in patients with type 2 diabetes mellitus with retinopathy. Mol Vis (2012) 18:219–26. doi: 10.1016/j.jcjo.2011.08.009 PMC3272054 22312190

[7] Huang J Yi H Zhao C Zhang Y Zhu L Liu B. Glucagon-like peptide-1 receptor (GLP-1R) signaling ameliorates dysfunctional immunity in COPD patients. Int J Chron Obstruct Pulmon Dis (2018) 13:3191–202. doi: 10.2147/COPD.S175145 PMC6186765 30349227

[8] American Diabetes Association. Clinical practice recommendations 2002. Diabetes Care (2002) 25 Suppl 1:S1–147. doi: 10.2337/diacare.25.2007.s1 11788484

[9] Wilkinson CP Ferris FL Klein RE Lee PP Agardh CD Davis M. Proposed international clinical diabetic retinopathy and diabetic macular edema disease severity scales. Ophthalmology (2003) 110:1677–82. doi: 10.1016/S0161-6420(03)00475-5 13129861

[10] Hummel CS Lu C Loo DD Hirayama BA Voss AA Wright EM. Glucose transport by human renal Na+/D-glucose cotransporters SGLT1 and SGLT2. Am J Physiol Cell Physiol 300 (2011) 300(1):C14–21. doi: 10.1152/ajpcell.00388.2010 PMC3023189 20980548

[11] Hebsgaard JB Pyke C Yildirim E Knudsen LB Heegaard S Kvist PH. Glucagon-like peptide-1 receptor expression in the human eye. Diabetes Obes Metab (2018) 20:2304–8. doi: 10.1111/dom.13339 PMC6099507 29707863

[12] Kim W Egan JM. The role of incretins in glucose homeostasis and diabetes treatment. Pharmacol Rev (2008) 60:470–512. doi: 10.1124/pr.108.000604 19074620 PMC2696340

[13] Abu-Hamdah R Rabiee A Meneilly GS Shannon RP Andersen DK Elahi D. Clinical review: The extrapancreatic effects of glucagon-like peptide-1 and related peptides. J Clin Endocrinol Metab (2009) 94:1843–52. doi: 10.1210/jc.2008-1296 PMC2690432 19336511

[14] Campbell JE Drucker DJ. Pharmacology, physiology, and mechanisms of incretin hormone action. Cell Metab (2013) 17:819–37. doi: 10.1016/j.cmet.2013.04.008 23684623

[15] Lin WJ Ma XF Hao M Zhou HR Yu XY Shao N. Liraglutide attenuates the migration of retinal pericytes induced by advanced glycation end products. Peptides (2018) 105:7–13. doi: 10.1016/j.peptides.2018.05.003 29746877

[16] Hao M Kuang HY Fu Z Gao XY Liu Y Deng W. Exenatide prevents high-glucose-induced damage of retinal ganglion cells through a mitochondrial mechanism. Neurochemistry Int (2012) 61:1–6. doi: 10.1016/j.neuint.2012.04.009 22542771

[17] Hernandez C Bogdanov P Corraliza L Garcia-Ramirez M Sola-Adell C Arranz JA. Topical administration of GLP-1 receptor agonists prevents retinal neurodegeneration in experimental diabetes. Diabetes (2016) 65:172–87. doi: 10.2337/db15-0443 26384381

[18] Yusta B Baggio LL Koehler J Holland D Cao X Pinnell LJ. GLP-1R agonists modulate enteric immune responses through the intestinal intraepithelial lymphocyte GLP-1R. Diabetes (2015) 64:2537–49. doi: 10.2337/db14-1577 25735732

[19] Taguchi M Someya H Inada M Nishio Y Takayama K Harimoto K. Retinal changes in mice spontaneously developing diabetes by Th17-cell deviation. Exp Eye Res (2020) 198:108155. doi: 10.1016/j.exer.2020.108155 32717339

[20] Manolescu AR Witkowska K Kinnaird A Cessford T Cheeseman C. Facilitated hexose transporters: new perspectives on form and function. Physiology (2007) 22:234–40. doi: 10.1152/physiol.00011.2007 17699876

[21] Wright EM Loo DD Hirayama BA. Biology of human sodium glucose transporters. Physiol Rev (2011) 91:733–94. doi: 10.1152/physrev.00055.2009 21527736

[22] DeFronzo RA Davidson JA Del Prato S. The role of the kidneys in glucose homeostasis: a new path towards normalizing glycaemia. Diabetes Obes Metab (2012) 14:5–14. doi: 10.1111/j.1463-1326.2011.01511.x 21955459

[23] Cani PD Holst JJ Drucker DJ Delzenne NM Thorens B Burcelin R. GLUT2 and the incretin receptors are involved in glucose-induced incretin secretion. Mol Cell Endocrinol (2007) 276:18–23. doi: 10.1016/j.mce.2007.06.003 17681422

[24] Gorboulev V Schurmann A Vallon V Kipp H Jaschke A Klessen D. Na(+)-d-glucose cotransporter SGLT1 is pivotal for intestinal glucose absorption and glucose-dependent incretin secretion. Diabetes (2012) 61:187–96. doi: 10.2337/db11-1029 PMC3237647 22124465

[25] Parker HE Adriaenssens A Rogers G Richards P Koepsell H Reimann F. Predominant role of active versus facilitative glucose transport for glucagon-like peptide-1 secretion. Diabetologia (2012) 55:2445–55. doi: 10.1007/s00125-012-2585-2 PMC3411305 22638549

[26] Gribble FM Williams L Simpson AK Reimann F. A novel glucose-sensing mechanism contributing to glucagon-like peptide-1 secretion from the GLUTag cell line. Diabetes (2003) 52:1147–54. doi: 10.2337/diabetes.52.5.1147 12716745

[27] Huang F Zhang L Song X Xu H Wang F Zhou L. The effects of different concentrations of glucose on glucose sensors and GLP-1 secretion in the enteroendocrine cell line STC-1. Gen Physiol biophysics (2020) 39:79–87. doi: 10.4149/gpb_2019040 32039827

[28] Powell DR Smith M Greer J Harris A Zhao S DaCosta C. LX4211 increases serum glucagon-like peptide 1 and peptide YY levels by reducing sodium/glucose cotransporter 1 (SGLT1)-mediated absorption of intestinal glucose. J Pharmacol Exp Ther (2013) 345:250–9. doi: 10.1124/jpet.113.203364 23487174

[29] Nakatsu Y Kokubo H Bumdelger B Yoshizumi M Yamamotoya T Matsunaga Y. The SGLT2 inhibitor luseogliflozin rapidly normalizes aortic mRNA levels of inflammation-related but not lipid-Metabolism-Related genes and suppresses atherosclerosis in diabetic ApoE KO mice. Int J Mol Sci 18 (2017) 18:1704. doi: 10.3390/ijms18081704 PMC5578094 28777298

[30] Garvey WT Van Gaal L Leiter LA Vijapurkar U List J Cuddihy R. Effects of canagliflozin versus glimepiride on adipokines and inflammatory biomarkers in type 2 diabetes. Metabolism (2018) 85:32–7. doi: 10.1016/j.metabol.2018.02.002 29452178

[31] Heerspink HJL Perco P Mulder S Leierer J Hansen MK Heinzel A. Canagliflozin reduces inflammation and fibrosis biomarkers: a potential mechanism of action for beneficial effects of SGLT2 inhibitors in diabetic kidney disease. Diabetologia (2019) 62:1154–66. doi: 10.1007/s00125-019-4859-4 PMC6560022 31001673

[32] Iannantuoni F Diaz-Morales MA,N Falcon R Banuls C Abad-Jimenez Z Victor VM. The SGLT2 inhibitor empagliflozin ameliorates the inflammatory profile in type 2 diabetic patients and promotes an antioxidant response in leukocytes. J Clin Med (2019) 8:1814. doi: 10.3390/jcm8111814 31683785 PMC6912454

[33] Wakisaka M Kitazono T Kato M Nakamura U Yoshioka M Uchizono Y. Sodium-coupled glucose transporter as a functional glucose sensor of retinal microvascular circulation. Circ Res (2001) 88:1183–8. doi: 10.1161/hh1101.091265 11397785

[34] Norton L Shannon CE Fourcaudot M Hu C Wang N Ren W. Sodium-glucose co-transporter (SGLT) and glucose transporter (GLUT) expression in the kidney of type 2 diabetic subjects. Diabetes Obes Metab (2017) 19:1322–6. doi: 10.1111/dom.13003 28477418

[35] Wang XX Levi J Luo Y Myakala K Herman-Edelstein M Qiu L. SGLT2 protein expression is increased in human diabetic nephropathy: SGLT2 PROTEIN INHIBITION DECREASES RENAL LIPID ACCUMULATION, INFLAMMATION, AND THE DEVELOPMENT OF NEPHROPATHY IN DIABETIC MICE. J Biol Chem (2017) 292:5335–48. doi: 10.1074/jbc.M117.779520 PMC5392679 28196866

[36] Fernandes R Hosoya K Pereira P. Reactive oxygen species downregulate glucose transport system in retinal endothelial cells. Am J Physiol Cell Physiol (2011) 300:C927–36. doi: 10.1152/ajpcell.00140.2010 21228321

[37] You ZP Zhang YL Shi K Shi L Zhang YZ Zhou Y. Suppression of diabetic retinopathy with GLUT1 siRNA. Sci Rep (2017) 7:7437. doi: 10.1038/s41598-017-07942-x 28785055 PMC5547104

[38] Badr GA Tang J Ismail-Beigi F Kern TS. Diabetes downregulates GLUT1 expression in the retina and its microvessels but not in the cerebral cortex or its microvessels. Diabetes (2000) 49:1016–21. doi: 10.2337/diabetes.49.6.1016 10866055

[39] Pardridge WM Triguero D Farrell CR. Downregulation of blood-brain barrier glucose transporter in experimental diabetes. Diabetes (1990) 39:1040–4. doi: 10.2337/diab.39.9.1040 2384187

[40] Marques C Mega C Goncalves A Rodrigues-Santos P Teixeira-Lemos E Teixeira F. Sitagliptin prevents inflammation and apoptotic cell death in the kidney of type 2 diabetic animals. Mediators Inflammation (2014) 2014:538737. doi: 10.1155/2014/538737 PMC4000968 24817793

